# Maternal mortality in Northeast Brazil 2009-2019: spatial distribution, trend and associated factors

**DOI:** 10.1590/S2237-96222023000300009.EN

**Published:** 2023-10-30

**Authors:** Ianne Vitória Gomes Oliveira, Thatiana Araújo Maranhão, George Jó Bezerra Sousa, Taynara Lais Silva, Maria Izabel Félix Rocha, Maria Madalena Cardoso da Frota, Thalis Kennedy Azevedo de Araujo, Maria Lúcia Duarte Pereira

**Affiliations:** 1 Universidade Estadual do Piauí, Curso de Enfermagem, Parnaíba, PI, Brazil; 2 Secretaria da Saúde do Estado do Ceará, Fortaleza, CE, Brazil; 3 Universidade Federal do Ceará, Programa de Pós-Graduação em Saúde Pública, Fortaleza, CE, Brazil; 4 Universidade Estadual do Ceará, Programa de Pós-Graduação em Cuidados Clínicos em Enfermagem e Saúde, Fortaleza, CE, Brazil

**Keywords:** Maternal Mortality, Ecological Studies, Spatial Analysis, Time Series Studies, Mortalidad Materna, Estudios Ecológicos, Análisis Espacial, Estudios de Series Temporales, Mortalidade Materna, Estudos Ecológicos, Análise Espacial, Estudos de Séries Temporais

## Abstract

**Objective:**

To analyze the spatio-temporal pattern of maternal mortality and associated factors in Northeast Brazil, from 2009 to 2019.

**Methods:**

This was an ecological study using the joinpoint method for temporal analysis and spatial autocorrelation and scan tests to identify clusters; regression models using the ordinary least squares and geographically weighted regression methods were used to identify factors associated with mortality, considering p-value < 0.05.

**Results:**

Maternal mortality decreased by 1.5% (95%CI; -2.5;-0.5) per year (p-value = 0.009); clusters, of deaths were found, mainly in Piauí and Maranhão, the variables associated with the maternal mortality ratio were Gini Index (β = 105.72; p-value < 0.001), municipal human development index (β = 190.91; p-value = 0.001), *per capita* income (β = -0.08; p-value = 0.001), Firjan Municipal Development Index-Health (β = -51.28; p-value < 0.001), life expectancy at birth (β = -3.50; p-value < 0.001).

**Conclusion:**

There was a reduction in mortality in the period studied, with a concentration of deaths, primarily in Piauí and Maranhão; socioeconomic indicators were associated with higher mortality in the region.

## Study contributions

**Main results:**

There was a decrease in maternal mortality in Northeast Brazil, from 2009 to 2019. Deaths were mainly concentrated in the states of Piauí and Maranhão. Five socioeconomic indicators were associated with higher mortality in the region.

**Implications for services:**

In order to maintain the trend of falling maternal mortality in Northeast Brazil, the need exists to reduce social inequalities and expand access to health services, especially within the scope of Primary Care.

**Perspectives:**

Public policies are needed to expand health services in general as well as comprehensive women’s health care in the Brazilian National Health System, especially for women living in contexts of greater social vulnerability.

## Resumen

**Objetivo:**

Analizar el patrón espacio-temporal y los factores asociados a la mortalidad materna en el Nordeste de Brasil, 2009-2019.

**Métodos:**

Estudio ecológico por el método joinpoint para análisis temporal y pruebas de autocorrelación espacial y escaneo scan para la identificación de conglomerados; se utilizaron modelos de regresión por mínimos cuadrados ordinarios y métodos geográficamente ponderados para identificar los factores asociados a la mortalidad, considerando p-valor < 0,05.

**Resultados:**

La mortalidad materna disminuyó 1,5% por año (IC_95%_ -2,5;-0,5; p-valor = 0,009), se observaron conglomerados de muertes en Piauí y Maranhão, variables asociadas a la tasa de mortalidad materna, Índice de Gini (β = 105,72; p-valor < 0,001), índice de desarrollo humano municipal (β = 190,91; p-valor = 0,001), renta per cápita (β=-0,08; p-valor = 0,001), Índice de Desarrollo Municipal-Salud de Firjan (β = -51,28; p-valor < 0,001), esperanza de vida al nacer (β = -3,50; p-valor < 0,001).

**Conclusión:**

Hubo una reducción de la mortalidad, las muertes se distribuyeron, principalmente, en Piauí y Maranhão; el aumento o la disminución de los indicadores socioeconómicos se asociaron a una mayor mortalidad en la región.

## Resumo

**Objetivo:**

Analisar o padrão espaço-temporal e fatores associados à mortalidade materna, no Nordeste brasileiro, de 2009 a 2019.

**Métodos:**

Estudo ecológico, com método *joinpoint*, para análise temporal, testes de autocorrelação espacial e varredura *scan* para identificar aglomerados, utilizando-se modelos de regressão pelos métodos de mínimos quadrados ordinários e de regressão espacial geograficamente ponderado, para identificar fatores associados (p-valor < 0,05).

**Resultados:**

A mortalidade materna diminuiu 1,5% ao ano (IC_95%_ -2,5;-0,5; p-valor = 0,009), observaram-se aglomerados de óbitos, principalmente nos estados do Piauí e Maranhão, variáveis associadas à razão de mortalidade materna foram índice de Gini (β = 105,72; p-valor < 0,001), índice de desenvolvimento humano municipal (β = 190,91; p-valor = 0,001), renda *per capita* (β = -0,08; p-valor = 0,001), Índice Firjan de Desenvolvimento Municipal-Saúde (β = -51,28; p-valor < 0,001) e esperança de vida ao nascer (β = -3,50; p-valor < 0,001).

**Conclusão:**

E mortalidade materna reduziu-se no período, com concentração de óbitos no Piauí e Maranhão; os indicadores socioeconômicos associaram-se a maior mortalidade na região.

## INTRODUCTION

Maternal death is defined as the death of women during the gestational period, childbirth and postpartum period, or up to 42 days after the outcome of pregnancy, regardless of the duration or location of pregnancy. Maternal death results from decisions made in relation to pregnancy, or from factors related to or aggravated by it, such as gestational hypertension, severe bleeding, infections, complications resulting from childbirth, unsafe abortions or problems associated with conditions acquired during pregnancy, such as malaria and human immunodeficiency virus (HIV) infection. These are factors the origin of which is found in obstetric causes, both direct and indirect, and not in accidental causes.^1^ ^),(^ ^2^

The maternal mortality ratio (MMR), expressed by the number of maternal deaths per 100,000 live births (LB) in a given location, is the indicator that, in addition to evaluating the quality of women’s health care, allows serial comparisons of this event to be carried out. Therefore, a high MMR is associated with low quality of women’s health care during the pregnancy-puerperal cycle.^3^

Furthermore, the MMR reflects living conditions - social, economic and cultural - that impact the health of a given population. The indicator’s relationship with these determinants is exemplified by data from Sub-Saharan Africa, where the planet’s lowest human development indices (HDIs) are found, as well as concentrating 70% of the world’s maternal deaths in 2020; and where, as one might expect, a very high MMR has been confirmed.^4^ ^),(^ ^5^ ^)^

One of the Millennium Development Goals (MDGs), established by the United Nations (UN) in 2000, was to reduce the MMR by 75% between 1990 and 2015. However, the majority of countries in the world did not meet this target; including Brazil, which reduced its MMR by just 57.7%.^6^ ^)^ In view of this, a new agenda of commitments was negotiated by the 193 UN member States and formalized on September 25, 2015: the 2030 Sustainable Development Agenda, which includes maternal health in its third Sustainable Development Goal (SDG), according to which countries should “meet the goal of reducing maternal mortality to less than 70 deaths per 100,000 live births by 2030”.^3^ This is a challenge which requires intense commitment ​​and investments by governments.

In Brazil, the Northeast region of the country stands out negatively. In 2020, only the state of Pernambuco had an MMR lower than the national average (74.7 deaths/100,000 LB). On the other hand, the states of Maranhão and Piauí had the highest coefficients in the region (109 and 101 deaths/100,000 LB, respectively).^7^ The fact that the states in the Northeast with the highest MMR are precisely those with the lowest *per capita* income in the country^8^ points to the influence of this socioeconomic determinant on the occurrence of maternal death.

The MMR is an indicator used in epidemiological analyses that measure trends and spatial inequalities in order to facilitate understanding of the dynamics of maternal mortality in a territory. Analysis of this coefficient also allows comparisons between municipalities, states and countries, either contributing to knowledge about the population’s socioeconomic performance and health levels, or serving as information for planning, management and evaluation of women’s health care strategies.^9^

The objective of this study was to analyze the spatio-temporal pattern of maternal mortality and associated factors in Northeast Brazil, from 2009 to 2019.

## METHODS

*Study design*

This was an ecological study, analyzing the temporal trend and spatial distribution of maternal mortality and its relationship with socioeconomic indicators, using municipalities as its units of analysis. According to the 2010 Demographic Census, carried out by the Brazilian Institute of Geography and Statistics (*Instituto Brasileiro de Geografia e Estatística* - IBGE), that year Northeast Brazil had 53,078,137 inhabitants, accounting for 28% of the country’s resident population, distributed over 1,794 municipalities in the region’s nine states: Alagoas, Bahia, Ceará, Maranhão, Paraíba, Pernambuco, Piauí, Rio Grande do Norte and Sergipe.^10^

*Participants*

We analyzed public domain secondary data on maternal deaths that occurred in the Northeast from 2009 to 2019. Data on these deaths were extracted from the website of the National Health System Department of Information Technology (*Departamento de Informática do Sistema Único de Saúde* - DATASUS) in November 2020. The information which supported the MMR calculations, available on the Mortality Information System (*Sistema de Informação sobre Mortalidade* - SIM) and on the Live Birth Information System (*Sistema de Informação sobre Nascidos Vivos* - SINASC), are recorded on Live Birth Certificates and Death Certificates.

*Variables*

The following variables were analyzed.

a) Maternal sociodemographic (categorical) variables:

- age group (at last birthday: 10-14; 15-19; 20-29; 30-39; 40-49; 50-59); 

- schooling (in completed years of study: none; 1-3; 4-7; 8-11; 12 or more);

- race/skin color (White; Asian; Black; mixed race; Indigenous; unknown);

- place of death (hospital; home; public thoroughfare; other health establishment; other; unknown); and

- marital status (single; married; widow; legally separated; other; unknown); 

b) Causes of death, as coded in Chapter XV (Complications in pregnancy, childbirth and the puerperium) of the Tenth International Statistical Classification of Diseases and Related Health Problems (ICD-10), corresponding to codes O00-O99, except codes O96 and O97, as well as codes B20-B24, D39.2, E23.0, M83.0, A34 and F53.

c) Socioeconomic indicators: 

- municipal human development index (*índice de desenvolvimento humano municipal* - IDHM), measures income, education and longevity indices, ranging from 0 to 1;

- Gini index of *per capita* household income, measures the level of income concentration, ranging from 0 to 1;

- life expectancy at birth (average number of years of life expected for a newborn);

- illiteracy rate (%) among people aged 18 or over;

- percentage (%) of people aged 18 or over with completed elementary education;

- percentage (%) of people vulnerable to poverty;

- *per capita* income in Brazilian Real (BRL); and 

- Firjan Municipal Development Index-Health (*Índice Firjan de Desenvolvimento Municipal-Saúde* - IFDM), Health element, retrieved from the website of the Rio de Janeiro State Industry Federation (*Federação de Indústrias do Estado do Rio de Janeiro* - Firjan), ranging from 0 to 1.^11^

Regarding filling in of the variables, completeness was used as an evaluation parameter. Completeness of an information system or a specific record category variable is good when greater than or equal to 75.1%; regular, when it varies from 75.0% to 50.1%; poor, from 50.0% to 25.1%; and very poor when equal to or less than 25.0%.^12^

*Data source and measurement*

The sociodemographic data on maternal deaths were obtained from the DATASUS website, while the demographic information and socioeconomic indicators of the municipalities resulted from consulting 2010 Demographic Census records.^10^ All socioeconomic indicators, as well as the absolute numbers of maternal deaths and the population in each year studied, were gathered into a single database, using Microsoft Office Excel software; and aggregated by municipality, with their respective geocodes. The socioeconomic indicators were accessed on the IBGE and DATASUS portals.

*Statistical methods*

Initially, the temporal trend of maternal mortality in the Northeast was analyzed. The raw death data, for each year of the series, were tabulated on an Excel spreadsheet and subsequently imported into the free Joinpoint Regression Program, version 4.6.0.0^®^, with the aim of achieving segmented linear analysis of the time series, also known as inflection point regression. This method allows testing whether one or more segments of the temporal trend curve, separated by inflection points, indicate a change in the mortality trend, opposing the null hypothesis that no point should be added to the model.^13^

The results of applying the Joinpoint Regression Program estimate the annual percentage change (APC) of the investigated trend, as well as its 95% confidence interval (95%CI), considering p-value < 0.05. To this end, the coefficients are calculated in the program itself, selecting the total number of deaths as the numerator and the LB population of the chosen year as the denominator, multiplied by 100,000 LB. Negative APC values point to a falling trend, while positive values indicate a rising trend; non-significant APC values (p-value > 0.05) indicate no variation in the trend, or stationary trend.^13^

Two types of rates were calculated and mapped to describe maternal mortality in municipalities. Initially, the average crude MMR was calculated using standardization via the indirect method: in the numerator, total number of maternal deaths for the period 2009-2019, divided by the total number of years studied (11 years), and in the denominator, number of LB of the population of each Northeastern municipality in the middle year (2014), multiplied by 100,000 LB.^14^ However, high crude rate instability in territories with small populations proved to be an obstacle. Thus, the smoothed MMR was calculated using the local empirical Bayesian method, which minimizes instability of the crude ratio values ​​and corrects random fluctuations. This method is important because it uses data from neighboring municipalities to predict the risk of an event in a given territory.^15^

The global Moran index was calculated in order to test the hypothesis of spatial dependence. Once global spatial autocorrelation was identified, the local Moran index was calculated [Local Index Spatial Analysis (LISA)] to identify spatial clusters and measure the degree of spatial association in each municipality. The results were represented by the Moran map, in which high/high and low/low patterns indicate points of positive spatial association, implying neighboring locations with similar values. The high/low and low/high patterns indicate points of negative spatial association, demonstrating neighboring locations with different values.^15^ ^),(^ ^16^ The LISA map confirms the statistical significance of the clusters, considering p-value < 0.05.^15^

The purely spatial Scan statistic technique was also used to identify spatial clusters and relative risk (RR) of mortality. RR values > 1 indicate that the mortality risk in the area is higher than the risk for the region as a whole.^15^

Finally, the analysis of socioeconomic indicators associated with maternal mortality was carried out using the ordinary least squares (OLS) non-spatial regression model. Then, the socioeconomic indicators that demonstrated statistical significance (p-value < 0.05) in the OLS model were input to a geographically weighted regression (GWR) model.^15^

The result of the GWR regression is presented on two thematic maps, for each socioeconomic indicator: one map for the value of the regression coefficient and the other map representing the statistical significance of each municipality, considering p-value < 0.05.^15^

The Akaike information criterion (AIC) and the coefficient of determination (R²) were considered as parameters for comparison between the OLS and GWR models and for choosing choice the one with the best fit: the lower the AIC value and the higher the R² value, the better the model fit.^15^

Using TerraView v.4.2.2^®^ software made it possible to calculate Bayesian statistics and spatial autocorrelation (global and local Moran indices). In the case of the Scan statistic, we used SatScan v.9.6^®^ software. Non-spatial OLS regression was performed using Stata 12^®^ software; while GWR spatial regression was performed using GWR4.0.9^®^ software. The maps were created using QGis v.2.14.17^®^ software.

*Ethical aspects*

As the study only used public domain secondary data, it was not necessary to submit the study project for appraisal by a Research Ethics Committee.

## RESULTS

A total of 6,232 maternal deaths were recorded in the Northeast region between 2009 and 2019. The largest proportion of these deaths corresponded to women aged 20 to 29 years (n = 2,517; 40.3%), of mixed race/skin color (n = 4,136; 66.3%), single marital status (n = 2,993; 48.0%) and with between 4 and 11 years of schooling (n = 3,329; 53.4%). The highest number of maternal deaths occurred in hospital (n = 5,566; 89.3%).

The main causes of maternal death were conditions classified elsewhere (n = 1,711; 27.8%) and edema, proteinuria and hypertensive disorders of pregnancy, childbirth and the postpartum period (n = 1,508; 24.5%). Regarding completeness of the variables, the greatest completeness was found in the “age group” variable (99.9%) and the lowest in the “schooling” variable (78.1%). Considering all variables, an average of 91.9% completeness was found, considered good (data not shown).

The average MMR, in the time series analyzed, was 68.0 deaths per 100,000 LB. Table 1 shows maternal mortality APC in the Northeast region and its respective states. The MMR for the entire region showed a significant decrease in mortality, namely 1.5% (95%CI -2.5;-0.5; p-value = 0.009) per year. All Northeastern states reported a falling or stationary MMR trend.

**Table 1:** Annual percentage change in maternal mortality in Northeast Brazil and respective states, 2009-2019

Region/state	Annual percentage change (95%CI^a^)	p-value^b^	Trend
Northeast	-1.5 (-2.5;-0.5)	0.009	Falling
Alagoas	-0.4 (-7.4;8.9)	0.907	Stationary
Bahia	-3.8 (-5.4;-2.1)	0.001	Falling
Ceará	-1.4 (-3.6;0.9)	0.194	Stationary
Maranhão	-1.0 (-3.6;1.8)	0.438	Stationary
Paraíba	2.3 (-1.7;6.4)	0.224	Stationary
Pernambuco	-0.4 (-2.7;2.0)	0.708	Stationary
Piauí	-1.6 (-3.7;0.6)	0.137	Stationary
Rio Grande do Norte	2.9 (-1.7;7.7)	0.193	Stationary
Sergipe	-6.6 (-9.4;-3.6)	< 0.001	Falling

a) 95%CI: 95% confidence interval; b) Permutation test.

**Figure 1:**
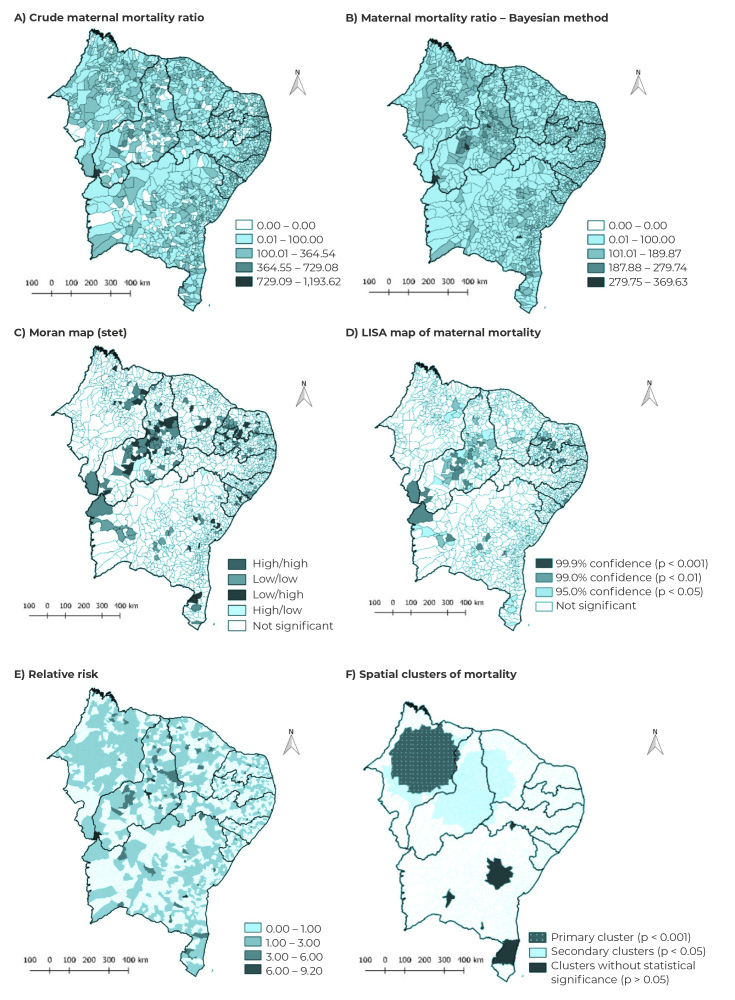
Crude maternal mortality ratio (A) and maternal mortality ratio using the Bayesian method (B), Moran map (C) and LISA map of maternal mortality (D), relative risk (E) and spatial clusters of maternal mortality (F), by municipalities of the states of Northeast Brazil, 2009-2019

The crude MMR map (Figure 1A) showed irregular distribution of rate values: a visible mosaic image, with a higher proportion of municipalities (n = 524) where the MMR was between 100.01 and 364.54 deaths per 100,000 LB. After smoothing, greater MMR stability was noted, with clusters of deaths predominantly in municipalities in the states of Piauí and Maranhão. The highest ratios (from 279.75 to 369.63 per 100,000 LB) were found in the Piauí municipalities of Barreiras do Piauí, Eliseu Martins and Tanque do Piauí (Figure 1B).

Significant global spatial autocorrelation was found (I = 0.04; p-value = 0.001). Classification of Northeast Brazilian municipalities on the Moran scatter plot showed that the high/high MMR pattern occurred mainly in Piauí; on the other hand, the low/low MMR pattern was found, in particular, in the far west of Bahia and in the west of Rio Grande do Norte (Figure 1C). The LISA map showed a high/high pattern of MMR distribution and statistical significance of the clusters (99.9%) in the municipalities of Piauí (Figure 1D). 

Figure 1E and F show the RR and the clusters of maternal mortality in the Northeast region, obtained by the Scan statistic. Most Northeastern municipalities had a RR for maternal mortality higher than the risk found for the region as a whole. Despite this, some municipalities located in the south of the state of Piauí (São José do Peixe, Tanque do Piauí and Barreiras do Piauí) stood out as having the highest RR values ​​in the Northeast (RR = 6.00 to 9.20) (Figure 1E). 

A total of 11 spatial clusters of maternal deaths were identified, of which only five were statistically significant (p-value < 0.05). The primary cluster, that is, the one that showed the lowest probability of the event occurring by chance, included 129 municipalities in Maranhão. The secondary clusters, which also proved to be statistically significant, were found in Maranhão, Piauí and Ceará (Figure 1F).

The primary cluster had a radius of 232 km and its municipalities had, on average, 1.48 time more risk of maternal deaths, compared to the risk of maternal deaths for the Northeast region as a whole (Table 2).

**Table 2:** Spatial clusters of maternal deaths defined by the purely spatial scan statistic in the states of Northeast Brazil, 2009-2019

Cluster	No. of municipalities	States	Radius (km)	No. of cases	Expected No. of cases	RR^a^	LLR^b^	p-value^c^
1	129	MA, PI	232.0	689	480.7	1.48	43.53	< 0.001
2	40	PI, MA, CE, PE	124.6	264	161.1	1.66	28.36	< 0.001
3	163	PI, MA, CE, PE	226.5	314	210.9	1.51	22.72	< 0.001
4	83	PI, MA, CE, PE	202.8	349	245.3	1.44	20.21	< 0.001
5	150	PI, MA, CE, PE	203.0	339	241.4	1.42	18.29	< 0.001
6	1	BA	0.0	22	8.0	2.75	8.25	0.238
7	30	BA	93.7	102	67.8	1.51	7.55	0.386
8	13	BA	107.0	95	62.8	1.52	7.20	0.475
9	2	BA	20.7	9	2.1	4.34	6.28	0.759
10	1	BA	0.0	9	2.3	3.93	5.61	0.921
11	1	BA	0.0	7	1.6	4.30	4.84	0.997

a) RR: Relative risk; b) LLR: Log-likelihood ratio; c) Scan statistic *p* test.

In the model OLS, the following indicators were statistically associated with MMR: Gini index [β = 105.72; 95%CI 52.85;158.59 (p-value < 0.001)], IDHM [β = 190.91; 95%CI 73.45;308.36 (p-value = 0.001)], *per capita* income [β = -0.08; 95%CI -0.13;- 0.04 (p-value = 0.001)], Firjan Municipal Development Index-Health [β = -51.28; 95%CI -70.37;-32.19 (p-value < 0.001)] and life expectancy at birth [β= -3.50; 95%CI -5.30;-1.69 (p-value < 0.001)] (Table 3). 

**Table 3:** Association between socioeconomic indicators and maternal mortality ratio, according to OLS and GWR regression models, Northeast Brazil, 2009-2019

Socioeconomic indicators	OLS^a^ Model			GWR^b^ Model		
	Coef.^c^	p-value^d^	95%CI^e^	Coef.^c^	Standard Error	95%CI^e^
Intercept	-	-	-	102.91	36.34	-9.07;304.36
Gini Index	105.72	< 0.001	52.85;158.59	112.92	26.28	-50.80;187.73
IDHM^f^	190.91	0.001	73.45;308.36	-0.41	0.10	-145.39;386.05
*Per capita* income	-0.08	0.001	-0.13;-0.04	-0.05	0.01	-0.13;0.03
IFDM^g^ - Health	-51.28	< 0.001	-70.37;-32.19	-1.11	0.92	-69.11;47.41
Life expectancy at birth	-3.50	< 0.001	-5.30;-1.69	-1.08	0.50	-4.5;0.68

a) Non-spatial ordinary least squares (OLS) regression model; b) GWR: Geographically weighted regression model; c) Coef.: coefficient; d) OLS regression p test; e) 95%CI: 95% confidence interval; f) IDHM: Municipal human development index (*índice de desenvolvimento humano municipal*); g) IFDM-Saúde: Firjan Municipal Development Index (*Índice Firjan de Desenvolvimento Municipal*) - Health.

In the OLS model, the coefficient of determination (R²) was 0.04 and the Akaike information criterion (AIC) was 19,452.52, while in the GWR model, the R² coefficient was 0.12 and AIC was 19,335.75. Thus, the GWR model proved to have a better fit because it presented a lower AIC and a higher R² and, in view of this, maps of the regression coefficients and statistical significance of each of the variables that were significant in the OLS model were built.

**Figure 2:**
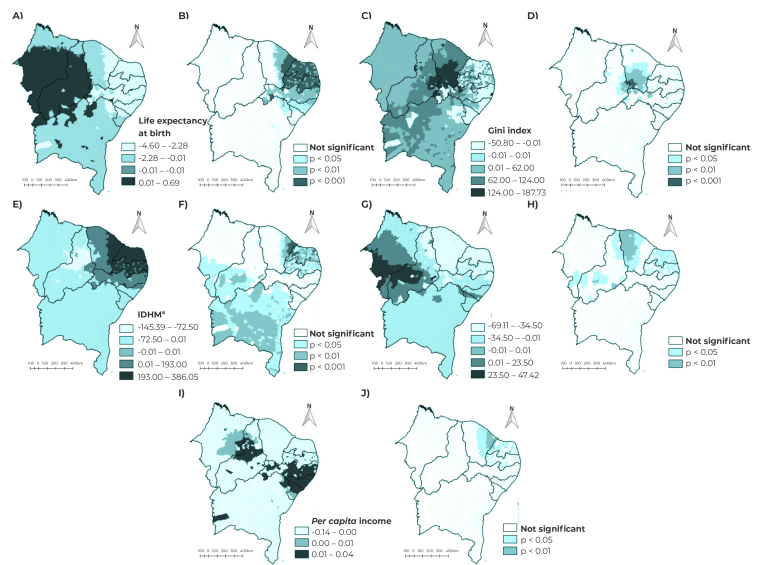
Spatial distribution of the maternal mortality ratio and of the statistical significance of the socioeconomic indicators in the GWR spatial model, Northeast Brazil, 2009-2019

Figure 2 shows the spatial distribution of the estimated coefficients and the statistical significance of the independent variables associated with maternal mortality in the Northeast, according to the GWR spatial regression model. Figure A and B show that the lower the life expectancy at birth, the higher the MMR in the states of Ceará, Rio Grande do Norte, Paraíba, Pernambuco and Alagoas. Furthermore, in municipalities located in the Sertão Cearense and southeast Piauí regions, there was positive association between MMRs and the Gini index, an indicator of social inequality, demonstrating that maternal mortality has increased as social inequality has grown in those territories (Figure 2C and D). 

In municipalities located in the Vale do Jaguaribe region of Ceará, Rio Grande do Norte and Paraíba, we found that the higher the municipal human development index, the higher the maternal mortality ratio. On the other hand, in Bahia and in municipalities in the south of Piauí and Maranhão, there was negative association of this indicator with maternal mortality (Figure 2E and F).

There was a significant negative association between the IFDM-Health and the dependent variable, in a significant portion of municipalities in Ceará, Rio Grande do Norte and Paraíba (maps G and H). Likewise, in municipalities in the Vale do Jaguaribe region of Ceará and in some municipalities in Rio Grande do Norte and Paraíba, the lower the *per capita* income, the higher the MMR (Figure 2I and J).

a) Municipal Human Development Index (*Índice de Desenvolvimento Humano Municipal* - IDHM); b) Firjan Municipal Development Index (*Índice Firjan de Desenvolvimento Municipal* - IFDM); GWR: Geographically weighted regression model.

## DISCUSSION

Throughout the period analyzed in this study, a falling MMR trend was found in the Northeast region. We identified that a significant portion of municipalities in the states of Piauí and Maranhão had the highest smoothed average rates of maternal mortality. These municipalities also stood out for having significant spatial clusters using the scan statistic, and clusters with a high/high pattern of distribution in the spatial autocorrelation test. It is also clear that the Gini index, municipal human development index, *per capita* income, life expectancy at birth and IFDM-Health are indicators associated with maternal mortality. 

Although a significant decrease in MMR was seen in the Northeast between 2009 and 2019, the number of maternal deaths and regional inequalities in the rates were still high and require more detailed analysis, with the aim of planning measures to reduce the event that address local demands and result in satisfactory indicators for maternal and child health.^17^ ^),(^ ^18^ ^)^

The Northeast region of Brazil presents considerable socioeconomic heterogeneity in its territory.^18^ Our analysis identified, especially in the sub-region located between the state of Maranhão and west Piauí, called the Mid-North, the municipalities with the worst poverty indicators, that is, high Gini indices and lower *per capita* income. It is worth highlighting that this sub-region presents low socioeconomic development, with a predominance of the primary sector: plant extractivism, traditional cotton, rice and sugar cane farming, in addition to extensive livestock farming, on the whole.^19^ ^)^

The low municipal human development index may be associated with the increase in MMR in municipalities in southwest Piauí, Maranhão and Bahia. This hypothesis is confirmed by a study that associates inadequate housing conditions, lack of basic sanitation, low level of schooling and socioeconomic precariousness with morbidity and mortality in the maternal population of Paraíba. ^(^ ^20^

Some municipalities in Piauí stood out for having the highest risk of maternal mortality in the Northeast. Based on Ministry of Health data, the high number of maternal deaths in these municipalities could be explained by shortcomings in Piauí’s health services. The main obstacles to achieving prenatal care include low adherence of health professionals to the use of risk stratification guides in prenatal care, difficulties in accessing and monitoring pregnant women, especially those at high risk, and lack of training of Primary Health Care professionals working in Brazilian National Health Service facilities.^21^

The results of this study reinforce the importance of socioeconomic indicators as predictors of maternal mortality in the region. We found that the higher the municipal human development index the higher the MMR in municipalities in Rio Grande do Norte, Paraíba and the Vale do Jaguaribe region of Ceará. Another study pointed out the existence of a correlation between the proportion of cesarean sections and the human development index, as more developed regions have higher rates of cesarean sections compared to poorer regions.^22^ Compared to normal childbirth, high rates of cesarean births are associated with serious complications for mothers, given that cesarean section is a surgical procedure that can pose greater maternal and perinatal risks.^23^ ^),(^ ^24^

Our analysis found that the greater the social inequality with regard to income, demonstrated here by the Gini index, the greater the MMR in the municipalities of the Ceará Sertão region. These territories are characterized by the frequency with which they are affected by drought, and by having a lower level of economic development. Poor communities with restricted access to health services report higher rates of maternal mortality, as low female purchasing power, associated with inaccessible health care, favors death from obstetric causes.^25^ ^),(^ ^26^

It was also found that the lower the *per capita* income, the higher the maternal mortality in municipalities in Rio Grande do Norte, Paraíba and Ceará. *Per capita* income is an important indicator of the degree of economic development of a region or country.^23^ For example, in Ecuador statistical association was found between gross domestic product (GDP) and the MMR in some provinces of that country, in addition to an estimate of 32 more maternal deaths per 100,000 LB in provinces that had lower GDP compared to those with higher GDP.^27^

In a considerable portion of municipalities in Ceará, significant negative association was found between IFDM-Health and maternal mortality. It is important to highlight that this indicator reflects the health care provided by primary health care centers.^12^ Similar results, also described in the literature, point to a relationship between socioeconomic disparities and a low number of prenatal consultations, in addition to reduced use of health centers and postnatal care services, with a direct impact on maternal death statistics.^28^

Negative association was found between the “life expectancy at birth” indicator and the MMR in the states of Ceará, Rio Grande do Norte, Paraíba, Pernambuco and Alagoas. A study on life expectancy in Brazil, Ethiopia and the United States highlighted that improvements in social determinants, such as income, education, housing, environment and infrastructure, contribute to the promotion of health equity and positively impact life expectancy at birth outcomes in those countries.^29^ Marked inequalities in health conditions reflect, in part, the economic and social inequalities of countries; therefore, richer nations, where living standards are higher and where investments in health-promoting factors, such as sanitation, housing and education, such as the United States, are greater, have much higher life expectancy when compared to poorer nations.^29^

The use of secondary data, derived from the SIM and the SINASC is one of the limitations of this study, due to inaccuracies in the records, resulting from inadequate data filling in, as well as underreporting. Also noteworthy is the fact that the indicators studied refer to the 2010 Demographic Census and therefore do not fully reflect the current socioeconomic status of the Northeast region and its states.

We conclude that maternal mortality showed a falling trend in the Northeast region of Brazil, and a greater concentration of maternal deaths in the states of Piauí and Maranhão. We also found association between the event and the socioeconomic indicators used, such as higher income concentration, poverty and lower life expectancy.

Promotion of maternal health goes beyond the basic aspects of pregnancy, childbirth and postpartum care. It also covers socioeconomic aspects, which influence women’s quality of life. We suggest that maternal mortality prevention strategies be targeted at municipalities with the highest occurrence of maternal deaths, highlighting the need to strengthen the Brazilian National Health System so that public reproductive health policies are improved and the right to women’s health is guaranteed.
